# Two new species of genus *Rhopalopsole* (Insecta, Plecoptera, Leuctridae) from China

**DOI:** 10.3897/zookeys.154.2234

**Published:** 2011-12-12

**Authors:** Qian Yu-Han, Du Yu-Zhou

**Affiliations:** 1 Institute of Applied Entomology, Yangzhou University, Yangzhou, Jiangsu 225009, China

**Keywords:** *Rhopalopsole*, Leuctridae, Plecoptera, new species, China

## Abstract

Two new species of *Rhopalopsole* Klapálek from China are described: *Rhopalopsole exiguspina* Du & Qian, **sp. n.** and *Rhopalopsole ampulla* Du & Qian, **sp. n.**, which were collected in Guizhou province, China.

## Introduction

The genus *Rhopalopsole* belongs to the family Leuctridae and is distributed throughout the Oriental and Palaearctic Regions. The abdominal segments of *Rhopalopsole* species are unmodified, but the last segment bears on both lateral sides a chitinous process, the shape of which is an important character for distinguishing species. Cerci are one-segmented and slightly modified in males, and cerci shape varies according to species (Kawai 1967). The genus *Rhopalopsole* first was described by Klapálek from a Taiwanese species, *Rhopalopsole dentata* Klapálek (1912). Contributions to and revisions of *Rhopalopsole* were made by Okamoto (1922), (Wu (1949, 1973), Illies (1966), (Kawai (1967, 1968, 1969), (Jewett (1958, 1975a, b), Harper (1977), Zwick (1977), Yang and Yang (1991a, b, 1994, 1995a, b), Yang et al. (2004) and Yang and Li (2006). Harrison and Stark (2008) provided a checklist of the genus that included 29 species, and an additional 43 species were subsequently described by Sivec et al. (2008), Stark and Sivec (2008), Yang et al. (2009), Li and Yang (2010), Li et al. (2010, 2011).

Here we describe two new *Rhopalopsole* species collected in Guizhou province, China. All type specimens were preserved in 75% ethanol and deposited in the Insect Collection of Yangzhou University, Jiangsu, China.

## Taxonomy

### Rhopalopsole exiguspina

Du & Qian, sp. n.

urn:lsid:zoobank.org:act:B26AB2A6-972F-4A18-9D1D-486A980CF80F

http://species-id.net/wiki/Rhopalopsole_exiguspina

Figs 1–4 

#### Material examined.

Holotype ♂ from China, Guizhou, Yanhe County, Shaba Village, 903m, 5 Oct. 2007, Leg. Xue Hai-Yang. Paratypes 18♂♂, the same details as holotype.

#### Adult habitus.

General color: Light brown. Head brown or light brown, wider than prothorax, hind ocelli much closer to the eyes than to each other, antennae and palpi yellowish brown. Prothorax light brown, subquadrate, all angles somewhat rounded and some black irregular stripes on it. Legs light brown. Wings hyaline and veins light brown.

#### Male.

**Figures 1–4.**
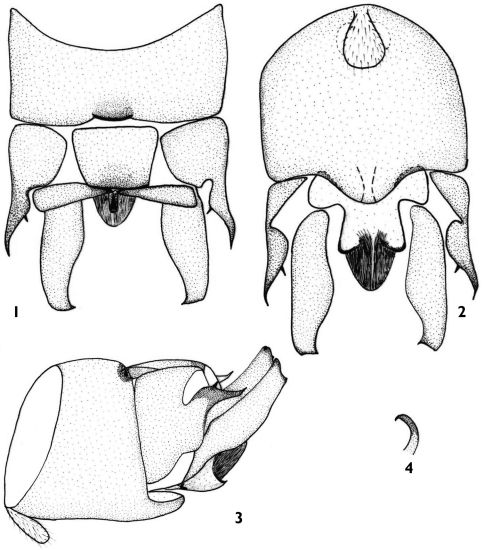
*Rhopalopsole exiguspina* male structures **1** Male terminal, dorsal aspect **2** Male terminal, ventral aspect **3** Male terminal, lateral aspect **4** Epiproct, lateral aspect.

Approximate measurement: forewing length 6.0 mm, body length 6.5 mm. Mid-posterior margins of tergite 9 sclerotized, slightly emarginated (Fig. 1). Sternite 9 basally with a tongue-like vesicle bears dense hairs, apically with a subgenital plate wider than long and rounded apically (Fig. 2). Tergite 10 with strongly sclerotized lateral process beak-like somewhat acute and curving inward apically and a small spine at the middle of lateral process in dorsal view, thick basally and slightly curved upward apically in lateral view. Mid-anterior sclerite sclerotized, posterior margin more sclerotized; one pair of transverse triangle sclerite weakly sclerotized (Fig. 1). Epiproct a simple curved process, erect hook-like apical portion curved inward (Fig. 4). Subanal lobe sinuate in lateral aspect, rounded and strongly sclerotized apically, apex membranous in ventral aspect; subanal lobe clearly with a pair of little lobes at middle of subanal lobe and each little lobes rounded apically in ventral aspect. Cerci long and cylindrical, thick basally and thin apically, distinctly upturned in lateral aspect, apex with a tiny spine.

#### Female.

Unknown.

#### Etymology.

The species name refers to the small spine at the middle of lateral process of tergite 10.

#### Diagnosis.

This new species resembles *Rhopalopsole aculeata* Harper (1977) from Nepal and *Rhopalopsole xui* Yang, Zhu & Li (2004) from Guangdong in having an epiproct with a thin spine-like apical portion and a strongly sclerotized lateral process without bifurcation of tergite 10. The new species can be distinguished from *Rhopalopsole aculeata* by the presence of spines on the lateral processes and cerci. *Rhopalopsole aculeate* has no spines on the middle of lateral process or on the cerci. The new species can be distinguished from *Rhopalopsole xui* by the shapes of the mid-anterior sclerite, lateral process, subanal lobe and cerci. In *Rhopalopsole xui*, the mid-anterior sclerite is wider than long and has two short obtuse lateral processes. *Rhopalopsole xui* lacks spines at the middle of the lateral processes and on the cerci and has a wide and apically rounded subanal lobe without a pair of small lobes. The characteristics of the subanal lobe and lateral process distinguish this new species from other *Rhopalopsole* species, which possess an epiproct with thin spine-like apical portions and no bifurcated lateral processes on tergite 10.

### Rhopalopsole ampulla

Du & Qian, sp. n.

urn:lsid:zoobank.org:act:B6B94919-A1E4-48D2-9CE6-6AA7A1DF63C9

http://species-id.net/wiki/Rhopalopsole_ampulla

Figs 5–7 

#### Material examined.

Holotype ♂ from China, Guizhou, Yanhe County, Shaba Village, 903m, 5 Oct. 2007, Leg. Xue Hai-Yang. Paratypes 6♂♂, the same details as holotype.

#### Adult habitus.

General color: Brown and dark brown. Head brown or dark brown, wider than prothorax, hind ocelli much closer to the eyes than to each other, antennae and palpi brown. Prothorax dark brown, quadrate, longer than wide, all angles rounded and some black irregular stripes on it. Legs light brown. Wings hyaline and veins light brown.

#### Male.

**Figures 5–7.**
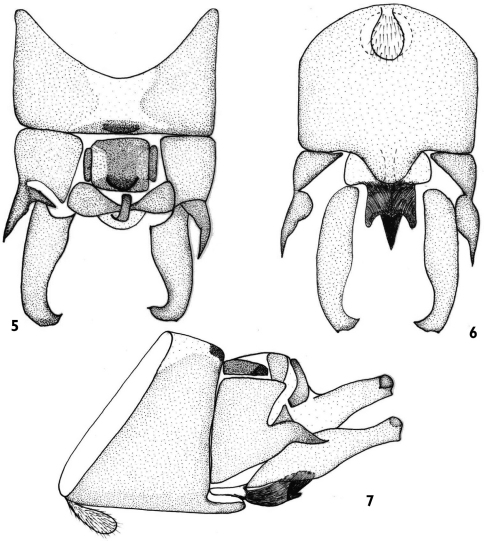
*Rhopalopsole ampulla* male structures **5** Male terminal, dorsal aspect **6** Male terminal, ventral aspect **7** Male terminal, lateral aspect.

Approximate measurement: forewing length 8 mm, body length 8.5 mm. Tergite 9 sclerotized, with a large central membranous area, the mid-posterior margin strongly sclerotized (Fig. 5). Sternite 9 with a subgenital plate wider than long and rounded apically, basally with a tongue-like vesicle bears dense hairs (Fig. 6). Tergite 10 with two small narrow lateral mid-anterior sclerites and one large broad median mid-anterior sclerite; mid-posterior more sclerotized and protrusive; one pair of transverse sclerite weakly sclerotized (Fig. 5). Lateral processes each strongly sclerotized, spine-like rather than thick basally, narrowed apically and downward in lateral aspect (Fig. 7). Epiproct curved forward, thick and blunt apically (Fig. 5, 7). Subanal lobe strongly sclerotized at base, trident-like apically in ventral aspect and membranous at its apex (Fig. 6). Cerci long and cylindrical, ampulla-like, thick basally and thin apically, each with a tiny spine.

#### Female.

Unknown.

#### Etymology.

The species name refers to the shape of cerci on segment 10.

#### Diagnosis.

This new species is similar to other species in the *Rhopalopsole assamensis* group (Sivec et al. 2008) in having a sclerotized area on the mid-posterior margin of tergite 9, thick epiproct, lateral sclerites at each side of the central sclerite and cerci with tiny spines. It can be diagnosed by the shape of the subanal lobes, which are trident-like apically. Other species in the *Rhopalopsole assamensis* group possess subanal lobes that are flat and narrow at the base but expand into a wide rectangular apical portion. The lateral processes of species in the *Rhopalopsole assamensis* group typically end in a forked process on tergite 10, but those of this new species lack bifurcation. *Rhopalopsole ampulla* is similar to *Rhopalopsole exiguspina*, but *Rhopalopsole ampulla* can be distinguished by the shapes of the subanal lobes and the lateral processes on tergite 10. The subanal lobes of *Rhopalopsole exiguspina* are rounded apically and each posses a small spine at the middle of lateral process, but those of *Rhopalopsole ampulla* are strongly sclerotized and trident-like apically in ventral aspect.

## Supplementary Material

XML Treatment for Rhopalopsole exiguspina

XML Treatment for Rhopalopsole ampulla
